# Thyroid metastasis from renal cell carcinoma presenting as a solid mass with rapid enlargement

**DOI:** 10.1530/EDM-23-0126

**Published:** 2024-01-31

**Authors:** Isabella Chiardi, Priska Gaffuri, Andrea Leoncini, Pierpaolo Trimboli

**Affiliations:** 1 Thyroid Unit of Clinic for Endocrinology and Diabetology, Lugano Regional Hospital, Ente Ospedaliero Cantonale, Bellinzona, Switzerland; 2 Faculty of Medicine and Surgery, Humanitas University, Rozzano, Milan, Italy; 3 Istituto Cantonale di Patologia, Ente Ospedaliero Cantonale, Bellinzona, Switzerland; 4 Servizio di Radiologia e Radiologia Interventistica, Istituto di Imaging Della Svizzera Italiana (IIMSI), Ente Ospedaliero Cantonale, Bellinzona, Switzerland; 5 Faculty of Biomedical Sciences, Università della Svizzera Italiana (USI), Lugano, Switzerland

**Keywords:** Adult, Female, White, Switzerland, Thyroid, Thyroid, Endocrine-related cancer, Error in diagnosis/pitfalls and caveats, January, 2024

## Abstract

**Summary:**

Thyroid metastases from nonthyroidal malignancies (NTMs) represent a diagnostic challenge, often displaying heterogeneous clinical manifestations. These metastases are rare but significant, accounting for approximately 2% of thyroid malignancies. Distinguishing them from primary thyroid malignancies is challenging due to the lack of specific ultrasound features, and the ultrasound-based risk stratification systems offer limited utility in such cases. Fine needle aspiration cytology is crucial for definitive diagnosis, yet it may not always provide accurate results. In this case report, we describe a unique instance of thyroid metastases originating from renal cell carcinoma, emphasizing the complexities in diagnosis and the importance of considering oncological conditions when assessing thyroid masses. Awareness of thyroid metastasis from NTMs, particularly in cases of diffuse thyroid hypoechogenicity and hypothyroidism, is essential for clinicians in their diagnostic approach.

**Learning points:**

- Thyroid metastases from nonthyroidal malignancies are diagnostic challenges due to their heterogeneous clinical presentations, often mimicking primary thyroid malignancies.
- Thyroid metastases from nonthyroidal malignancies are relatively rare, but they still account for approximately 2% of thyroid malignancies.
- It is fundamental to consider oncological conditions when assessing thyroid masses, especially in cases of diffuse thyroid hypoechogenicity, hypothyroidism, and history of other tumors.
- Thyroid presentation is quite similar to that of autoimmune hypothyroidism, endocrinologists must be aware of the possibility of thyroid hypofunction due to the massive invasion of the parenchyma.

## Background

Thyroid metastases from nonthyroidal malignancies (NTMs) are uncommon and represent a significant diagnostic challenge for clinicians. In fact, they often exhibit a diverse and heterogeneous manifestation. Despite the unimpaired thyroid gland's function, it can become a battleground for the silent thyroid parenchymal invasion of NTM. While relatively rare, thyroid metastases from NTMs were found in 2.3–7.5% of patients submitted to fine needle aspiration cytology (FNAC) and this scenario is estimated to account for approximately 2% of all thyroid malignancies (1, 2). It is important to note that thyroid metastases constitute a separate clinical entity from primary thyroid malignancies. However, distinguishing between them is quite challenging, as there are no specific ultrasound (US) features to rely on (3). Therefore, a comprehensive understanding of the patient’s medical history and clinical presentation is essential before finally confirming the diagnosis on cytological samples.

In this case report, we present an intriguing clinical scenario of thyroid metastasis from a renal cell carcinoma (RCC), highlighting the complexities surrounding their recognition and diagnosis. RCCs primarily affect adults and are most observed during the sixth decade of life, with a higher incidence among males. The metastatic spread of RCC typically occurs in various sites, including the lower respiratory tract, skeletal system, lymph nodes, brain, liver, and skin, with occasional involvement of other locations, such as the thyroid. Although secondary involvement of the thyroid by RCC is infrequent, it remains one of the more prevalent NTM to metastasize to the thyroid gland. This tumor is very unpredictable, and its metastases could sometimes present the initial manifestation of the disease, mimicking, for example, a primary thyroid gland tumor or an autoimmune hypothyroidism, thereby posing a complex diagnostic evaluation for clinicians. Moreover, studies have shown that in cases where a solitary metastasis from RCC is found in any anatomical site, the 5-year survival rate following nephrectomy is significantly higher compared to cases with widespread disease. Consequently, when such solitary metastatic foci are identified in the thyroid gland, surgical resection should be considered to potentially give a more favorable clinical outcome (4).

Thus, reporting the clinical presentation and history of a patient with massive thyroid metastasis from RCC achieves interest for many clinicians, such as endocrinologists, oncologists, urologists, radiologists, pathologists, and surgeons.

## Case presentation

**Figure 1:**
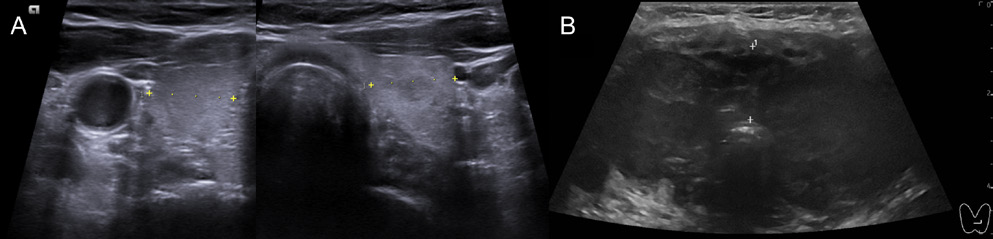
Thyroid US presentation in 2022 (A) and 1 year later (B). The complete changes of the echo-structure and the rapid increase of thyroid size are impressive.

We present the case of a 76-year-old female who had previously visited our endocrinology department in 2022. At that time, the indication for the visit was a biochemical asymptomatic thyrotoxicosis with suppressed TSH and mild increase of free T4, with negative anti-thyroid antibodies. Thyroid US performed during that visit revealed a homogeneous echo-structure and a volume within the normal range for her gender and weight (Fig. 1A). This patient’s medical history included a diagnosis of RCC in December 2015, specifically the papillary type with Fuhrman grade 3, staged as T2a R0, and successfully treated with a radical left nephrectomy. In 2020, metastatic lesions were identified in both pleura and mediastinal lymph nodes. With this clinical history, considering the biochemical data, and according to thyroid US presentation and the absence of specific symptomatology, it was suggested to proceed with laboratory follow-up to confirm the normalization of thyroid hormones. Really, the thyroid function was rapidly restored in a month. Subsequently, the patient developed chronic renal disease, leading to the initiation of dialysis in April 2023. During these years, the patient was also diagnosed with ischemic cardiopathy with preserved ejection fraction, hypertension, and recurrent urinary infections as comorbid conditions.

## Investigation

**Figure 2:**
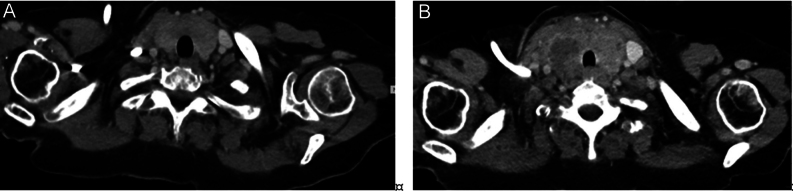
(A) CT-scan performed at the end of July with contrast agent shows the thyroid gland slightly enlarged with slight inhomogeneity of the structure. (B) CT-scan performed with contrast agent, 5 days later, shows the enlarged thyroid gland with a nonhomogeneous structure, with hypodense areas, which surrounds the trachea.

**Figure 3:**
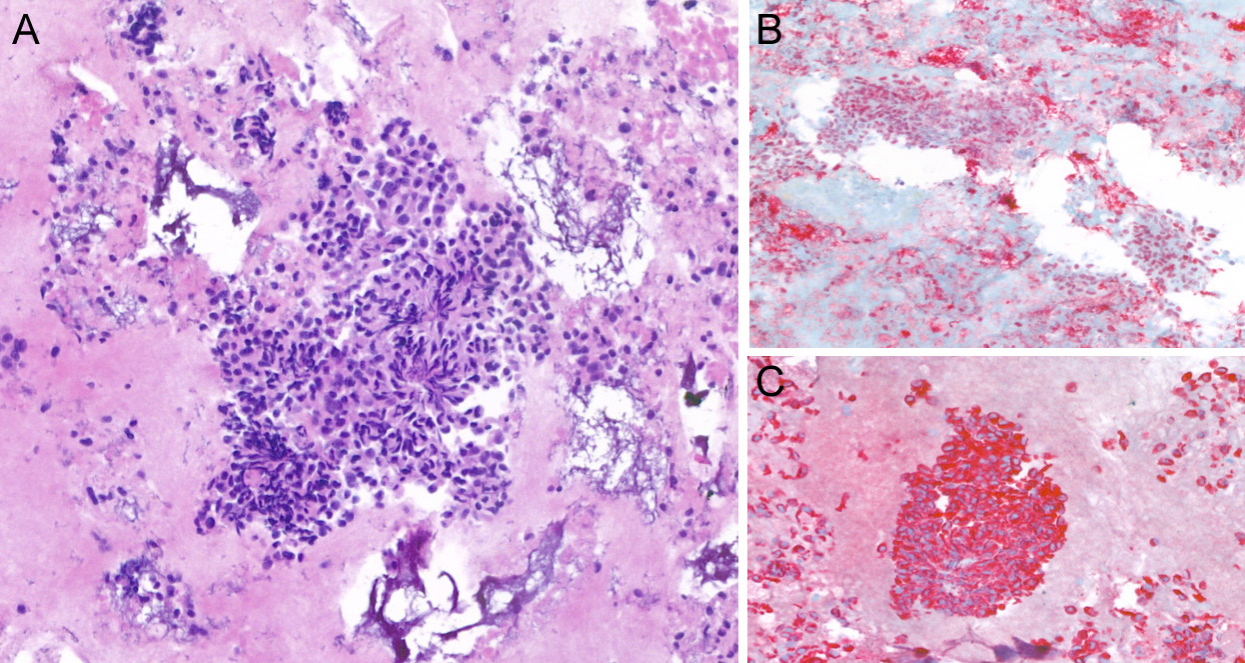
(A) The formalin-fixed, paraffin-embedded cell block sections showing isolated or in papillary structures cells with enlarged nuclei, with granular chromatin and moderately represented cytoplasm. (B and C) Immunohistochemistry showing strong, diffuse nuclear immunoreactivity for PAX8 and cytoplasmic immunoreactivity for CK19 in the tumor (A, hematoxylin–eosin-stained cell block, original magnifications 20×; B and C, original magnifications 20×).

In July 2023, in CT scan performed during the annual oncological follow-up, the neck region was initially described as showing signs of inflammation, with thyroid involvement (Fig. 2). In addition, the patient reported pain and mild compressive local symptoms, but despite receiving antibiotics and corticosteroids, she experienced no benefit. At that time thyroid function tests were normal except TSH, which was mildly elevated (4.9 mIU/L). However, in August, thyroid laboratory results revealed TSH: 111.1 mIU/L, free T3: 1.8 pmol/L, and free T4: 3.3 pmol/L. In response to these findings, the patient started treatment with levothyroxine. Subsequently, a thyroid US was performed, which revealed a conspicuous hypoechoic mass encompassing the entire thyroid gland (Fig. 1B). Cytological analysis demonstrated the presence of malignant cells suspicious for metastatic lesion originating from the RCC (Fig. 3). Then, it was concluded for hypothyroidism due to the massive disruption of thyroid parenchyma.

## Discussion

In our case, the malignancy affecting the thyroid was identified as a metastasis originating from RCC. It has been reported that RCC is the most common cancer metastasizing to the thyroid gland, being responsible for almost half (i.e. 48.1%) of thyroid metastasis from NTMs. Other tumors that metastasize to the thyroid are lung, colorectal, and breast carcinomas. Moreover, these studies have showed that clinical manifestations happen in around 75% of cases, mainly presenting with a new neck mass, neck swelling and dysphagia, like in our case. However, 25.1% of thyroid metastases from NTM are incidentally discovered on physical examination or imaging (5). In general, as mentioned before, the thyroid is not a common site for tumor spread, and this could be surprising due to the extensive blood supply of the thyroid. It has been suggested that metastasis development may be influenced by the glandular microenvironment; the fast arterial blood flow and the high concentration of oxygen and iodine may prevent the growth of circulating tumor cells (6).

Metastatic RCC can mimic primary thyroid gland neoplasms, potentially leading to diagnostic difficulties. Diagnosing thyroid metastases proves to be difficult due to their highly heterogeneous behavior, a problem shared with follicular and medullary thyroid carcinoma. In fact, relying solely on US features is insufficient to distinguish between primary and metastatic thyroid malignancies (3). This is because no single US feature offers enough sensitivity and specificity to reliably determine whether thyroid nodules are benign or malignant (7). However, the use of the Thyroid Imaging Reporting and Data System (TIRADS) can help to identify suspicious lesions. Unfortunately, TIRADSs were conceived based on the US presentation of papillary thyroid carcinoma, their reliability was nearly exclusively tested against that cancer, and thyroid metastases from NTM were very rarely observed in studies evaluating TIRADS performance (8). In general, US imaging has shown that most thyroid metastases typically appear homogeneously hypoechoic, with indistinct margins, irregular shapes, and increased vascularity in most cases (7). In our case, as observed in the US image (Fig. 1B), the thyroid exhibited marked hypoechoic characteristics, involving the entire thyroid tissue resulting in overt hypothyroidism. This presentation was notably different from a typical thyroid nodule. In fact, considering the significantly elevated TSH values, we also considered an autoimmune hypothyroidism as a potential differential diagnosis. From this point of view, nodular thyroid metastases may present heterogeneously according to US and TIRADSs (3). When looking at the CT findings of our case, we can affirm that, even if the 2023 presentation was significantly different from the previous one, CT scan cannot help to discriminate between primary cancer, metastasis from other organs, and other. Therefore, the true metastatic nature of the tumor can only be definitively recognized through tumor sampling and subsequent cytological assessment.

During FNAC, the cytological examination performed on the formalin-fixed, paraffin-embedded cell block sections revealed the presence of malignant cells, isolated or with papillary architecture showing nuclear–cytoplasmic atypia. Additionally, these cells tested positive for PAX8 and CK19 and were negative for TTF1. The diagnosis was positive for malignant cells with a commentary; the morphologic and immunocytochemical appearance appear most indicative for a localization of known renal primitiveness. Even if this was not the case, it is noteworthy to recognize that cytological assessment may sometimes fail to accurately diagnose metastatic nodules, as demonstrated in the study by Chung *et al.* (5), where FNAC did not provide the correct diagnosis in approximately 28.7% of patients with RCC. Actually, it was studied that the most difficult morphological diagnoses concern renal cell and breast carcinomas. These tumors may show an alveolar/glandular structure resembling the follicular pattern observed in thyroid hyperplastic nodules, necessitating the need for immunohistochemical techniques (2). Additionally, negative staining with anti-thyroglobulin and anti-calcitonin antibodies can be a valuable tool in favoring a diagnosis of metastatic tumor (5). This distinction could help differentiate it from primary thyroid malignancies, emphasizing the importance of comprehensive diagnostic approaches when dealing with thyroid nodules in patients with a history of malignancy for accurate differentiation and assessment.

Massive thyroid metastases from RCC, such as the one presented here, represent a clinical dilemma for physicians. They occur very rarely and both oncologists and urologists may not always be fully aware of this potential risk. Thyroid metastases have a very rapid evolution and patients present often with no specific local symptoms. Neck US is not included in the follow-up of RCC cases. Radiologists may become involved when whole-body imaging (i.e. CT scan and MRI) is indicated by oncologists or urologists, but these procedures cannot provide specific data about the malignancy risk. Molecular imaging (i.e. ^18^F FDG PET/CT) can be inconclusive due to the low glucose metabolism of kidney cancer cells. Performing biopsy may be tricky because of the hardness of the metastatic tissue. Cytological examination may be problematic due to the distinction between thyrocytes and metastatic cells. Immunohistochemical and molecular evaluations may not always be univocal in favor of RCC. Since the thyroid presentation is quite similar to that of autoimmune hypothyroidism, endocrinologists must be aware of the possibility of thyroid hypofunction due to the massive invasion of the parenchyma. This is an important issue that highlights the significance of a physical examination by endocrinologists, combined with careful collection of clinical history and neck US performed during the visit.

With this complex clinical scenario, the major recommendation in patients suspected for thyroid metastasis is to make maximum effort to achieve its rapid diagnosis, independently of the primary tumor. In fact, the enlarging thyroid mass can lead to death with choking. Thus, achieving rapidly the diagnosis is critical to potentially plan thyroidectomy. This operation may improve the prognosis and can avoid dramatic death. In any case, a multidisciplinary discussion is essential to ensure the best possible management and outcomes for these patients.

## Declaration of interest

The authors declare that there is no conflict of interest that could be perceived as prejudicing the impartiality of the case study reported.

## Funding

This case study received no external funding. The APC was funded by Università della Svizzera Italiana (USI).

## Patient consent

The patient signed the general institutional consent for retrospective data analysis; thus, there was no need for further specific consent.

## Author contribution statement

Conceptualization: PT; resources: PT; data curation: IC, PG, AL; writing – original draft preparation: IC; writing – review and editing: IC, PG, AL, PT. All authors have read and agreed to the final version of the manuscript.
